# EphA8 is a prognostic marker for epithelial ovarian cancer

**DOI:** 10.18632/oncotarget.8018

**Published:** 2016-03-09

**Authors:** Xiaoqin Liu, Yunzhao Xu, Qin Jin, Wei Wang, Shu Zhang, Xudong Wang, Yuquan Zhang, Xujuan Xu, Jianfei Huang

**Affiliations:** ^1^ Department of Nursing, Nantong University, Nantong 226001, Jiangsu, China; ^2^ Department of Obstetrics and Gynecology, Nantong University Affiliated Hospital, Nantong 226001, Jiangsu, China; ^3^ Department of Pathology, Nantong University Affiliated Hospital, Nantong 226001, Jiangsu, China; ^4^ Department of Laboratory Medicine, Nantong University Affiliated Hospital, Nantong 226001, Jiangsu, China

**Keywords:** EphA8, human epithelial ovarian cancer (EOC), immunohistochemistry, qRT-PCR, prognosis

## Abstract

EphA8 is one of the Eph receptors in the Eph/ephrin receptor tyrosine kinase (RTK) subfamily. During tumorigenesis, EphA8 is involved in angiogenesis, cell adhesion and migration. In this study, we determined the mRNA and protein expression levels of EphA8 in cancerous and normal ovarian tissue samples by quantitative reverse transcription PCR (qRT-PCR) (*N* = 60) and tissue microarray immunohistochemistry analysis (TMA-IHC) (*N* = 223) respectively. EphA8 protein levels in cancer tissues were correlated with epithelial ovarian cancer (EOC) patients’ clinical characteristics and overall survival. Both EphA8 mRNA and protein levels were significantly higher in EOC tissues than in normal or benign ovarian tissues (all *P* < 0.05). High EphA8 protein level was associated older age at diagnosis, higher FIGO stage, positive lymph nodes, presence of metastasis, positive ascitic fluid, and higher serum CA-125 level. High EphA8 protein level is an independent prognostic marker in EOC. We conclude that EphA8 acts as an oncogene in EOC development and progression. Detection of EphA8 expression could be a useful prognosis marker and targeting EphA8 represents a novel strategy for EOC treatment.

## INTRODUCTION

Epithelial ovarian cancer (EOC) is the seventh most common cancer and the eighth most common cause of cancer death in women worldwide. There were approximately 239,000 new cases diagnosed and 159,000 women died of EOC in 2012 (GLOBOCAN 2012). Although China has lower incidence rate of EOC [1] compared to western countries, the burden of EOC is not decreasing, instead the incidence in rural regions is predicted to be rising [2]. Because of the lack of specific symptoms during early stages of the disease, about 75% of EOC cases are diagnosed at advanced stage (stage III and IV) [3]. Further, the location of ovary hinders the development of morphology-based screening methods. Despite recent intensive research on the identification of biomarkers for early detection, currently, the only clinical available blood biomarker for EOC screening is CA125, which suffers from low sensitivity and specificity [4]. The standard treatment for women with advanced stage EOC is surgical cytoreduction followed by systemic chemotherapy [5]. Recent developments on EOC targeted therapies include angiogenesis inhibitors and poly (ADP-ribose) polymerase (PARP) inhibitors [6–7]. Despite these advances, the overall survival of EOC patients has not been significantly improved [8]. Novel markers for diagnosis and prognosis as well as new therapeutic targets are needed.

Ephrin receptors (Ephs) and ephrins are the largest subfamily of receptor tyrosine kinases (RTKs) [9]. Both are membrane-bound proteins that possess a unique capacity to initiate bi-directional intercellular downstream signaling pathways following cell-cell contact in both Eph-bearing (forward signaling) and ephrin-bearing cells (reverse signaling) [10]. During embryonic development, Eph-ephrin signaling pathway is involved in axon guidance, formation of tissue boundaries, cell migration and segmentation. In adulthood, they are involved in the maintenance of long-term potentiation, angiogenesis, and stem cell differentiation [11]. Ephs and ephrins display complex expression patterns in both cancer cells and tumor stroma cells, and they are implicated in multiple aspects of cancer development and progression, including tumor growth, migration/invasion, tumor stem cells, angiogenesis and metastasis [12–13]. In human, there are 14 Eph receptors (EphA1-8, EphA10, EphB1-4, EphB6) and eight ephrins (ephrin-A1-5, ephrin-B1-3) [14–15].

EphA8 functions as a receptor for GPI-anchored ephrin-A2, A3 and A5. When activated by ephrin-A5, phosphorylated EphA8 regulates integrin-mediated cell adhesion and migration on fibronectin substrate as well as neurite outgrowth and axon guidance through downstream FYN and MAP kinase signaling pathways [16]. During early brain development, EphA8 induces apoptosis in a caspase-dependent manner in ephrin-A5+ cells [17]. Downregulation of EphA8 has been detected in colon cancer as well as glioblastoma [18]. In glioma, downregulation of EphA8 by miR-10a induces epithelial mesenchymal transition (EMT) to promote tumor migration and invasion [19]. In EOC, aberrant expression of various Ephs and ephrins has been reported and associated with tumor aggressiveness and overall survival [20–26]. However, no studies have reported the expression of EphA8 in EOC.

In the current study, we determined both mRNA and protein expression of EphA8 in EOC tissue samples by quantitative reverse transcription PCR (qRT-PCR) and tissue microarray immunohistochemistry analysis (TMA-IHC) respectively, and correlated to patients’ clinical characteristics and overall survival.

## RESULTS

### EphA8 mRNA level was significantly higher in EOC tissues than in normal and benign ovarian tissues

**Figure 1:**
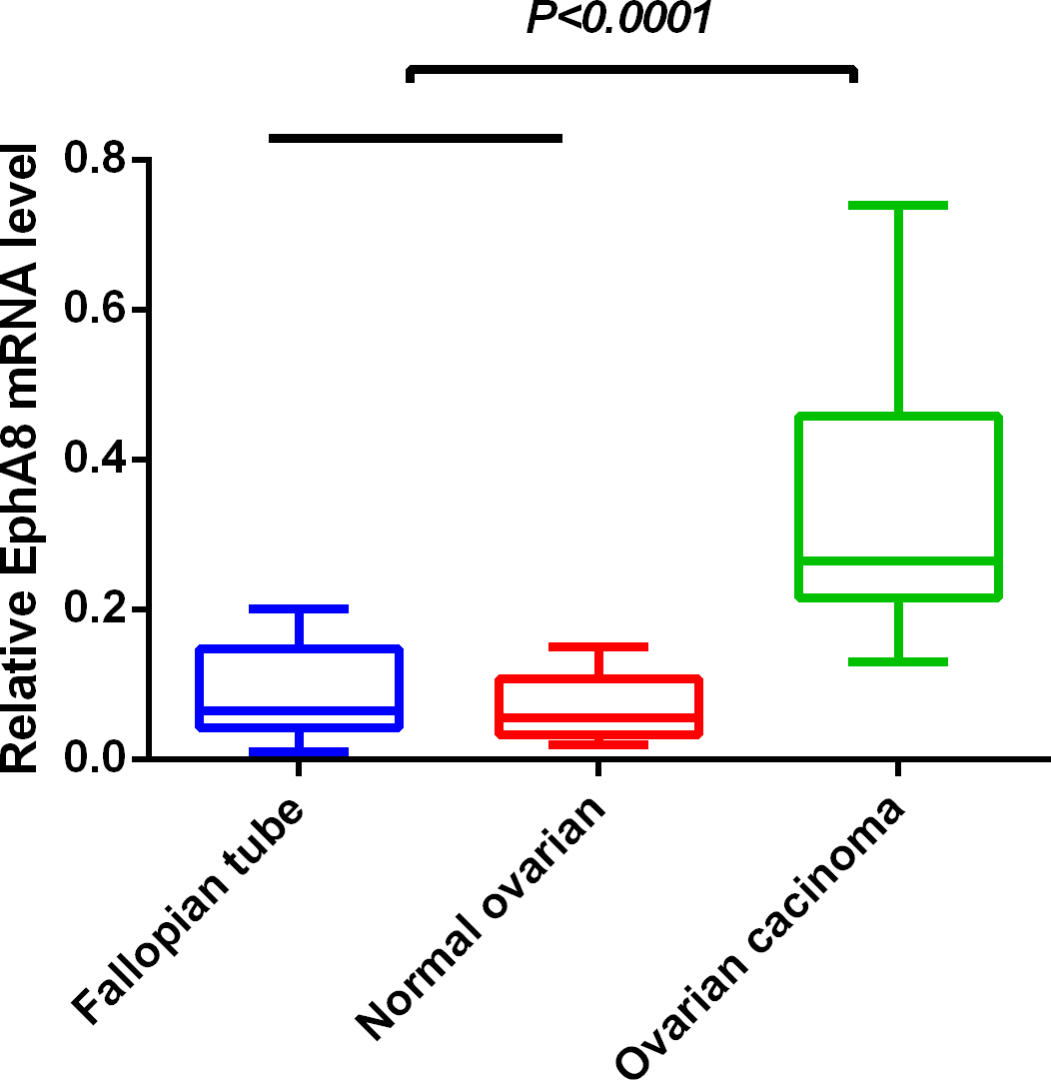
EphA8 mRNA level was significantly higher in ovarian cancer tissues than in normal fallopian tube and normal ovarian tissues EphA8 mRNA was determined by qRT-PCR and relative quantification analysis by normalizing to β-actin mRNA.

We determined EphA8 mRNA level in 60 fresh frozen ovarian tissues, including 20 normal fallopian tube samples, 20 normal ovarian tissues, and 20 EOC tissues. Relative EphA8 expression level was normalized to the expression of housekeeping gene β-actin. EphA8 mRNA expression level was significantly higher in EOC tissues (0.342 ± 0.038) when compared to normal fallopian tube tissues (0.087 ± 0.013) and normal ovarian tissues (0.071 ± 0.010) (*P* < 0.001) (Figure 1).

### EphA8 protein level was significantly higher in EOC tissues than normal and benign ovarian tissues

**Figure 2:**
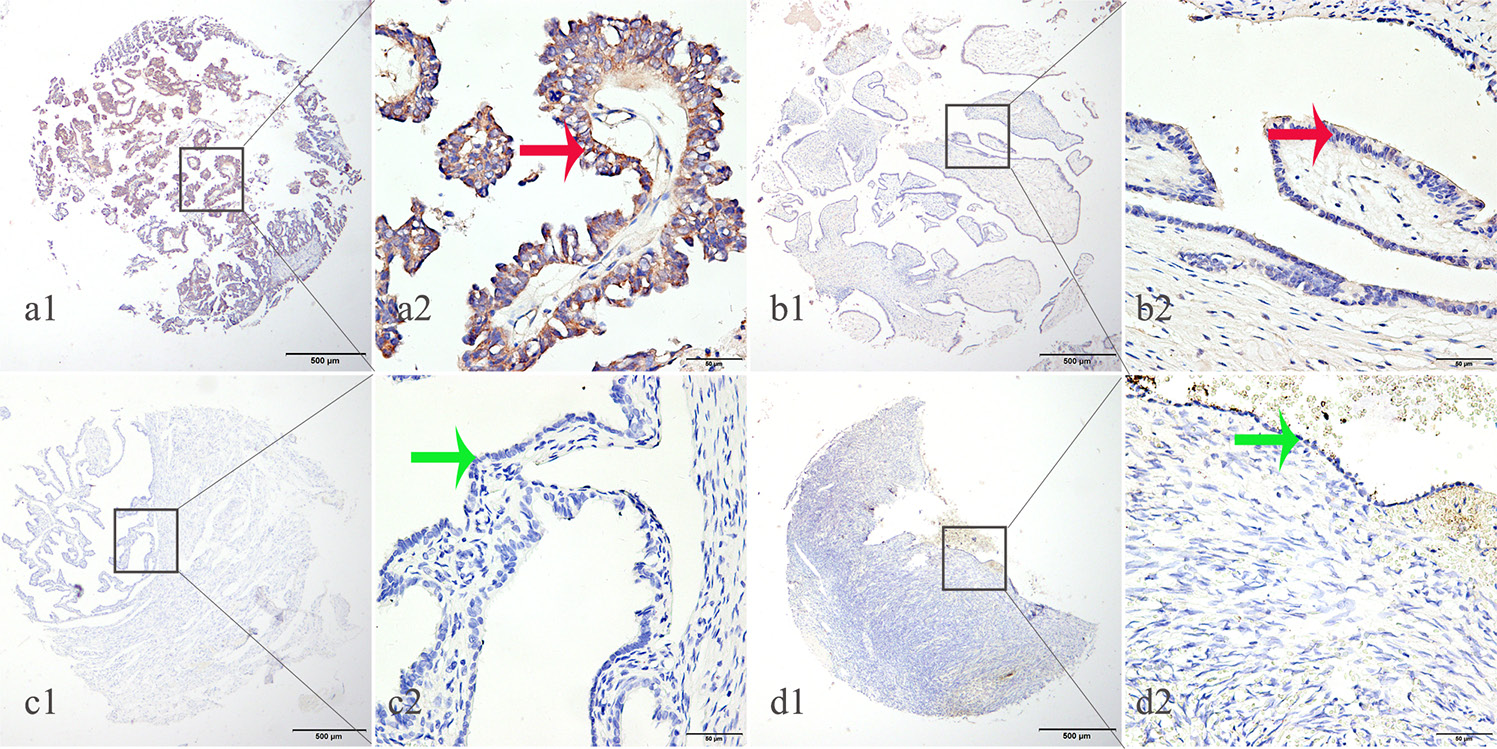
EphA8 protein was detected in ovarian cancer tissues but not in normal fallopian tube and normal ovarian tissues EphA8 protein was determined by TMA-IHC, (**a1**–**a2**) ovarian serous papillary carcinoma, strong positive for EphA8 protein expression; (**b1**–**b2**) ovarian serous adenoma, weak positive for EphA8 protein expression; (**c1**–**c2**) normal fallopian tube tissues, negative for EphA8 protein expression; (**d1**–**d2**) normal ovarian tissues, negative for EphA8 protein expression. a1, b1, c1 and d1 are ×40 magnification (bar = 500 μm), a2, b2, c2 and d2 are ×400 magnification (bar = 50 μm). Red arrows indicate positive EphA8 protein expression on cancerous epithelial cell membranes, and green arrows indicate negative EphA8 protein expression on normal ovarian epithelial cell membranes.

We subsequently determined EphA8 protein expression in 223 archived ovarian tissue blocks, including 125 EOC tissues, 30 borderline ovarian tumor tissues, 30 benign ovarian tumor tissues, 20 normal fallopian tube tissues, and 18 normal ovarian tissues. Epithelial EphA8 expression was analyzed for each tissue block: high EphA8 expression was detected in 44.80% of EOC tissues, but only detected in 6.67%–15% of normal or benign ovarian tissues (Table 1, Figure 2). The frequency of high EphA8 expression in EOC tissues was significantly higher than in normal and benign ovarian tissues (Pearson *χ* ^2^ = 31.962, *P* = 0.001).

**Table 1:** Immunohistochemical staining of EphA8 protein in normal ovarian, normal fallopian tube, benign ovarian tumor, borderline ovarian tumor and EOC tissues

Tissue sample	*n*	EphA8 expression			
		Low or none	High	Pearson *χ*	*P*- value
Normal ovarian tissue	18	16 (88.89)	2 (11.11)	31.962	0.001*
Normal fallopian tube tissue	20	17 (85.00)	3 (15.00)		
Benign ovarian tumor	30	28 (93.33)	2 (6.67)		
Borderline ovarian tumor	30	27 (90.00)	3 (10.00)		
EOC	125	69 (55.20)	56 (44.80)		

### Association of EphA8 expression with EOC clinical characteristics

Next, we correlated EphA8 protein expression with EOC patients’ clinical characteristics. High EphA8 protein expression was significantly associated with older age (60 years, *P* = 0.002), higher stage (FIGO stage II–IV, *P* = 0.001), presence of metastasis (*P* = 0.001), positive ascetic fluid (*P* = 0.047), and higher serum CA-125 level (> 100 U/ml, *P* = 0.038) (Table 2).

**Table 2:** Correlation of EphA8 protein expression with EOC patients’ clinicopathologic characteristics

Groups	EphA8				
	*n* = 125	Low or no (*n* = 69)	High (*n* = 56)	Pearson c^2^	*P*-value
Age at diagnosis				9.969	0.002*
60 years	75	50 (66.67)	25 (33.33)		
60 years	50	19 (38.00)	31 (62.00)		
FIGO stage				19.677	0.001*
mI	61	46 (75.41)	15 (24.59)		
II–IV	64	23 (35.94)	41 (64.06)		
Histological classification				0.067	0.967
Serous carcinoma	97	53 (54.64)	44 (45.36)		
Endometrioid carcinoma	16	9 (56.25)	7 (43.75)		
Other^a^	12	7 (58.33)	5 (41.67)		
Grade				2.688	0.101
Low	36	24 (66.67)	12 (33.33)		
High	89	45 (50.56)	44 (49.44)		
Positive lymph node				3.833	0.05
No	102	60 (58.82)	42 (41.18)		
Yes	20	7 (35.00)	13 (65.00)		
Unknown	3	2	1		
Metastasis				23.641	0.001*
No	68	51 (75.00)	17 (25.00)		
Yes	57	18 (31.58)	39 (68.42)		
Positive ascetic fluid				3.941	0.047*
No	61	35 (57.38)	26 (42.62)		
Yes	31	11 (35.48)	20 (35.48)		
Unknown	33	23	10		
Double or single				2.764	0.096
No	77	47 (61.04)	30 (38.96)		
Yes	48	22 (45.83)	26 (54.17)		
Serum CA-125 (U/ml)				4.312	0.038*
≤ 100	15	12 (80.00)	3 (20.00)		
> 100	103	53 (51.46)	50 (48.54)		
Unknown	7	4	3		

* *P* < 0.05 indicates a significant association among the variables; Metastasis: pelvic lymph node metastases or nearby tissues and organs involved.

a, others: clear cell carcinoma, 3 cases; mucinous carcinoma, 5 cases; transitional cell carcinoma, 2cases; adeno-squamous carcinoma, 2 cases.

### High EphA8 protein expression predicts poor overall survival in EOC patients

**Figure 3:**
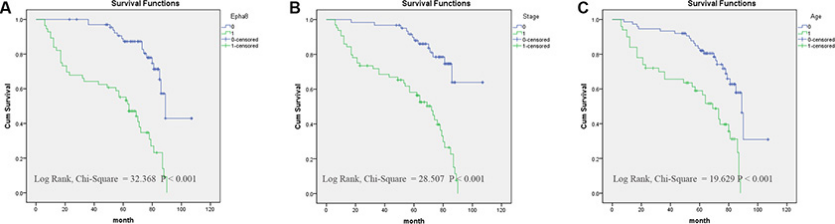
Survival curves of EOC patients by the Kaplan–Meier method and the log-rank test (**A**) EphA8+ EOC patients (green line, 1) had significantly worse overall survival than EphA8- patients (blue line, 0); (**B**) EOC patients with advanced stage (FIGO II–IV stage) (green line, 1) had significantly worse overall survival than patients with early stage (FIGO I stage) (blue line, 0); (**C**) EOC patients diagnosed at older age (60) (green line, 1) had significantly worse overall survival than patients diagnosed at younger age (< 60) (blue line, 0).

Finally, we analyzed prognostic factors in EOC patients using both univariate and multivariate analysis. In univariate analysis, we identified following prognostic markers associated with poor overall survival: higher EphA8 expression (HR, 4.614, 95% CI: 2.598–8.193; *P* = 0.001), older age at diagnosis (HR, 3.181, 95% CI: 1.848–5.475; *P* = 0.001), higher FIGO stage (HR, 4.651, 95% CI: 2.496–8.665; *P* = 0.001), higher tumor grade (HR, 2.026, 95% CI: 1.063–3.863; *P* = 0.032), positive lymph nodes (HR, 2.084, 95% CI: 1.131–3.843; *P* = 0.019), and presence of metastases (HR, 4.869, 95% CI: 2.694–8.801; *P* = 0.001). Because lymph node positivity and metastasis are already considered in the FIGO stage, all these significant factors except these two factors were included in the subsequent multivariate analysis. In multivariate analysis, higher EphA8 expression (HR, 2.591, 95% CI: 1.376–4.877; *P* = 0.003), older age at diagnosis (HR, 1.925, 95% CI: 1.084–3.420; *P* = 0.025), and higher FIGO stage (HR, 2.412, 95% CI: 1.195–4.869; *P* = 0.014) remained significantly associated with poor overall survival (Table 3). Similar results were shown by the Kaplan-Meier survival curve analysis (log rank, *P* < 0.001, Figure 3).

**Table 3:** Prognostic markers for overall survival in EOC patients by univariate and multivariate Cox proportional hazard model analysis

Variable	Univariate analysis			Multivariate analysis		
	HR	*P*-value	95% CI	HR	*P*-value	95% CI
EphA8						
High vs. low	4.614	0.001*	2.598–8.193	2.591	0.003*	1.376–4.877
Age (years)						
< 60 vs. ≥ 60	3.181	0.001*	1.848–5.475	1.925	0.025*	1.084–3.420
FIGO Stage						
II–IV vs. I	4.651	0.001*	2.496–8.665	2.412	0.014*	1.195–4.869
Histological type						
Sc vs. Ec vs. Others	.649	0.080	0.399–1.053			
Grade						
Low vs. High	2.026	0.032*	1.063–3.863	1.377	0.352	0.702–2.704
Positive lymph node						
Yes vs. No	2.084	0.019*	1.131–3.843			
Positive ascitic fluid						
Yes vs. No	1.534	0.144	0.864–2.722			
Metastasis						
Yes vs. No	4.869	0.001*	2.694–8.801			
Double or single						
Yes vs. No	1.118	0.684	0.655–1.907			
Serum CA-125 (U/ml)						
< 100 vs. ≥ 100	2.580	0.113	0.800–8.320			

Sc, serous carcinoma; Ec, endometrioid carcinoma;

HR: Hazard ratio; CI: Confidence interval.

* *P* < 0.05.

## DISCUSSION

In this study, we determined mRNA and protein expression levels of EphA8 in both malignant and normal ovarian tissues. EphA8 mRNA level was significantly higher in ovarian cancer tissues than in normal ovarian tissues or normal fallopian tube tissues. Similarly, EphA8 protein level was significantly higher in ovarian cancer tissues than in normal ovarian tissues, benign ovarian tumors and borderline tumors. High EphA8 protein level was associated with higher age at diagnosis, higher FIGO stages, presence of metastasis, positive ascetic fluid, and higher serum CA125 level. Finally, high EphA8 protein expression is an independent prognostic marker for poor overall survival in EOC patients.

The Eph/ephrin signaling pathway plays multifaceted roles in tumorigenesis and cancer progression. Eph/ephrin can act as oncogenes in human cancer. Several Eph receptors and ephrins are upregulated in a wide variety of cancer types [27]. In fact, the first Eph receptor (EphA1) and the first ephrine (ephrin-A1) were both identified as tumor antigens from carcinoma cell lines [28–29], and their overexpression could lead to oncogenic transformation in NIH3T3 fibroblasts [30]. Both gene amplifications and mutations of Eph receptors have been identified in human cancers [31–33]. Finally, overexpression of Eph receptors is linked to poor clinical outcome and cancer progression [34–35]. Mechanistically, upregulation of Eph/ephrin is associated with angiogenesis and tumor vasculature, including breast, lung, and prostate cancer, melanoma and leukemia [36–37]. Because Eph/ephrin signaling pathways modulate diverse processes during normal embryonic and adulthood development, including cell-cell interaction and cell migration, upregulation of Eph/ephrin promotes tumor growth, tumor stem cells, epithelial mesenchymal transition (EMT), invasion and metastasis, thus is associated with the more aggressive tumor behavior [36, 38]. Paradoxically, Eph/ephrin can also act as tumor suppressors: their expression is downregulated either through promoter methylation or loss of heterozygosity in several types of human cancer [39–43]. Both Eph forward and reverse signaling can contribute to tumor suppression. In cancer cells, Eph forward signals are silenced, and ephrins expressed in the adjacent normal tissues inhibits tumor expansion and invasiveness [27, 44].

In EOC, both overexpression and silencing of Ephs and ephrins have been observed. Overexpression of ephrins A1 and A5 in EOC was associated with poor survival [26]; Ephrin B1 expression was associated with high-grade carcinomas and microvessel density as well as higher rates of disease recurrence and poor overall survival [25]; EphB4/ephrinB2 expression level was increased with increased clinical stages and higher EphB4/ephrinB2 expression was associated with poor survival [24]; and silencing both EphA2 and EphB2 by siRNA has synergetic antitumor effect [22]. On the other hand, decreased expression of EphB6 or EphB1 was associated with high-grade EOC, metastasis and poor outcome [20–21].

EphA8 is the receptor for ephrin A2, A3 and A5, and plays an essential role in short-range contact-mediated axonal guidance during mammalian nervous system development [45–46]. EphA8 also promotes integrin-mediated cell adhesion and migration through PI3-kinase and MAPK kinase signaling pathways during normal embryonic development [16, 47]. It has been shown that EphA8 is downregulated in colon cancer and glioblastoma [18], and EphA8 expression is downregulated by miR-10a to promote migration and invasion through EMT in glioma [19]. The current study is the first to demonstrate EphA8's involvement in EOC and the first to show EphA8 is upregulated in cancer.

Our study has several limitations. First, it is a retrospective study, thus subject to sample selection bias as well as the availability of clinical data. Our results on EphA8 in EOC need to be confirmed in future studies involving larger number of EOC cases. Second, we did not perform laser microdissection to isolate ovarian epithelial cells for the mRNA expression analysis, thus both epithelial cells and stroma cells can contribute to the expression of EphA8. However, our IHC analysis suggests that majority of EphA8 expression is from epithelial cells. Finally, we did not provide the mechanistic insight of EphA8 in ovarian cancer development and progression. *In vitro* studies are needed to determine the function of EphA8 in EOC before EphA8 and its ligands could be considered as potential therapeutic targets in EOC.

In conclusion, our study demonstrates the involvement of EphA8 in EOC development and as an independent prognostic marker for EOC. We also provide evidence for targeting EphA8 and its ligands signaling pathways as novel EOC therapies. Both *in vitro* mechanistic studies and *in vivo* prospective studies are required to confirm and extend our conclusions. Our data support the development of novel cancer treatment strategies exploiting Eph/ephrin signaling pathways in EOC.

## MATERIALS AND METHODS

### Human tissue specimens and patient clinical information

EOC patients consent, enrollment, clinical data and sample collections were carried out as described before [48]. Briefly, we used 223 formalin-fixed paraffin-embedded (FFPE) tissue blocks and 60 fresh frozen tissue samples in the study. There were 18 patients with normal ovarian tissue, 20 patients with normal fallopian tube tissue 30 patients with benign ovarian tumors, 30 patients with borderline ovarian tumors, and 125 patients with EOC. All ECO patients received standard surgery and platinum-based chemotherapy after resection for 6–8 cycles. None of the patients received any therapy (chemotherapy, radiotherapy, or immunotherapy) prior to surgery. Patients were followed for 120 months right after surgery. Of the 125 cases of ovarian cancer, there were 97 of serous carcinoma, 16 of endometrial carcinoma, and 12 of other types (3 clear cell carcinoma, 5 mucinous carcinoma, 2 transitional cell carcinoma, and 2 adeno-squamous carcinoma). There were 61 stage I, and 64 stage II–IV cases. As for histological grading, 97 cases were high grade and 16 low grade. All these Patient clinical data were recorded in detail in Table 2. The study protocol was approved by the Human Research Ethics Committee of the Affiliated Hospital of Nantong University, Jiangsu, China.

### EphA8 expression and statistical analysis

EphA8 mRNA level was determined by quantitative reverse transcription PCR (qRT-PCR) [48]. The primers for EphA8 are as follows: forward primer (5′-CCA CCA GGG TAT GTA AAT ATC-3′) and reverse primer (5′-TGT GCT TTG AAG ACC ATT T-3′). EphA8 protein expression in tissue blocks was determined using tissue microarray immunohistochemistry (TMA-IHC) [48]. Rabbit polyclonal anti-human EphA8 antibody was used (dilution 1:40, HPA031433, Atlas, Sweden). The EphA8 IHC data were scored using the semi-quantitative H-score method taking into account both the staining intensity and the percentage of cells at that intensity [49], ranging from 0–300. Subsequently, the continuous EphA8 protein expression data were converted into dichotic data (low vs high) using specific cutoff values, which were selected to be significant in terms of overall survival (OS) using the X-tile software program (The Rimm Lab at Yale University; http://www.tissuearray.org/rimmlab) [49, 50–51]. In the current study, the cutoff was 100: score 0–100 was considered low expression while 101–300 was considered high expression.

Statistical analysis was performed as described before [48]. Student *t* test was used to compareq RT-PCR data between normal and tumor samples. *χ*^2^ tests were performed to determine the correlation between EphA8 expression and clinicopathologic parameters. Univariate and multivariate Cox regression models were used to identify prognostic factors. Kaplan-Meirer method was used to calculate survival curves. For all analyses, a *P*-value < 0.05 was regarded as statistically significant. Data were analyzed using SPSS 20 statistics software (SPSS Inc., Chicago, IL, USA) and STATA 12.0 (StataCorp, College Station, TX, USA).
